# Supplementary material for: Comparison of Indexes to Measure Comorbidity Burden and Predict All-Cause Mortality in Rheumatoid Arthritis
Source: J Clin Med. 2021 Nov 22;10(22):5460. doi: 10.3390/jcm10225460 (PMC8618526; doi:10.3390/jcm10225460)
Supplement: Supplementary file 1 [file jcm-10-05460-s001.zip › jcm-1442492-supplementary.pdf]

Table S1. Comparison of comorbidity indexes in patients with rheumatoid arthritis.

Author, year of publication (Ref)	Country	Study design	No. of RA patients	Comorbidity index	Summary of the study
England et al., 2015 (20)	US<br><br>Veterans Affairs Rheumatoid Arthritis (VARA) registry and the Rheumatology and Arthritis Investigator's Network Database (RAIN-DB)	Cohort	4765	RDCI, CDI, FCI, ETS, EPS, and COUNT	ETS and RDCI best predicted death. The FCI best predicted function
Radner et al., 2015 (18)	US<br><br>Brigham and Women's Rheu- matoid Arthritis Sequential Study(BRASS) and COMOrbidities in Rheumatoid Arthritis Study(COMORA)	Cohort	876	MMI.count, MMI.weight, MMI.weight using beta-coefficients, CCI, and FCI	MMI based on EQ-5D performed better than CCI. Not much improvement was obtained by weighting.
Putrik et al., 2018 (25)	Norway<br><br>Norwegian Register-DMARD study (patients with inflammatory joint diseases treated with DMARDs)	Cohort	4126	RDCI, CDI, FCI	Three indexes performed comparably well. CDI performing slightly worse when predicting functioning and health.
Nikiphorou et al., 2019 (26)	UK<br><br>The Royal College of General Practitioners (RCGP) Research and Surveillance Centre (RSC) database	Cohort	6591	CCI, RDCI	In early RA, both the RDCI and CCI were associated with an increased risk of all-cause mortality. RDCI predicts all-cause mortality better than CCI.
England et al., 2021(27)	US<br><br>Commercial insurance database(MarketScan)	Cohort	138891	CCI, RDCI	CCI and RDCI showed a higher burden of multimorbidity in RA. Similar findings were obtained when requiring ≥1 year of follow-up after RA diagnosis.

Abbreviations: Rheumatic Disease Comorbidity Index (RDCI) with the Charlson-Deyo Index (CDI), functional comorbidity index (FCI), Elixhauser Total Score (ETS), Elixhauser Point System (EPS), simple comorbidity count (COUNT), counted multimorbidity index (MMI.count), weighted multimorbidity index (MMI.weight), MMI.weight using beta-coefficients (weighted multimorbidity index using beta-coefficients), Charlson comorbidity index (CCI).

Table S2. The formula of four comorbidity indexes.

Index	Number of conditions	Formula
Dartmouth-Manitoba's CCI (range 0–33)	17	6 × (metastatic solid tumor + acquired immune deficiency syndrome)<br>+3 × (severe or moderate liver disease)<br>+2 × (hemiplegia + renal disease + diabetes with chronic complications + any malignancy, including leukemia and lymphoma)<br>+1 × (myocardial infarction + congestive heart failure + peripheral vascular disease + cerebrovascular disease + dementia + chronic obstructive pulmonary disease + connective tissue disease + ulcer + mild liver disease + diabetes, mild to moderate)

Elixhauser point system (range -19 to 89)	30	12 × metastatic cancer<br>+11 × liver disease<br>+9 × lymphoma<br>+7 × (congestive heart failure + paralysis)<br>+6 × (weight loss + other neurological disorder)<br>+5 × (cardiac arrhythmias + renal failure + fluid or electrolyte disorder)<br>+4 × (pulmonary circulation disorder + solid tumor without metastasis)<br>+3 × (chronic pulmonary disease + coagulopathy)<br>+2 × peripheral vascular disease<br>+0 × (acquired immune deficiency syndrome + alcohol abuse + diabetes, complicated + diabetes, uncomplicated + hypertension + hypothyroidism + peptic ulcer disease + psychoses + RA/collagen vascular disease)<br>- 1 × valvular disease<br>- 2 × (blood loss anemia + deficiency anemia)<br>- 3 × depression<br>- 4 × obesity<br>- 7 × drug abuse
Counted MMI (range 1–12)	12	1 × (hypertension + obesity + depression + cancer + diabetes mellitus + asthma + coronary heart disease + viral hepatitis + chronic obstructive pulmonary disease + chronic kidney disease + diverticulitis + stroke)
RDCI (range 0–9)	11	2 × lung disease + [2 × (heart attack, other cardiovascular disease, or stroke) or 1 × hypertension] + fracture + depression + diabetes + cancer + (ulcer or stomach problem)

Table S3. Comparison of the different comorbidity indexes.

	CDI	ECI	MMI	RDCI
Number of diseases	19	30	40	11
Original outcome	One-year mortality	Hospital stay, cost, and mortality	Quality of life	Mortality, hospitalization, disability, and costs
Unique disease	Dementia	Alcohol related disease, obesity, and thyroid disorders	Blindness, dementia, alcohol related disease, obesity, and thyroid disorders	Fracture
Common disease	Lung disease, stroke, cancer, heart failure, diabetes, coronary heart disease			
Number of papers about rheumatoid arthritis before 2018	Numerous	2	1	2
Dementia	O	X	O	X
Epilepsy	X	O	O	X
Liver disease	O	O	O	X
Lung disease	O	O	O	O
Blindness	X	X	O	X
Renal disease	O	O	O	X
Substance/alcohol use	X	O	O	X
Depression	X	O	O	O
Heart failure	O	O	O	O
Obesity	X	O	O	X
Thyroid disorder	X	O	O	X
Stroke	O	O	O	O
Cancer	O	O	O	O
Diabetes	O	O	O	O
Connective tissue disease	O	O	X	X

O Comorbidity which is included in the comorbidity index. X Comorbidity which is not included in the comorbidity index

Table S4. Characteristics of matched control group and comorbidity prevalence.

Characteristic	Value				
N	99,068				
Male, n(%)	20,560 (20.8)				
Age years, mean±SD	50.1±15.7				
Comorbidity indexes, mean±SD					
CCI	0.6±1.3				
ECI	1.7±4.2				
MMI	0.5±0.9				
RDCI	0.8±1.3				
Place of residence, n(%)					
Urban	57,975 (58.5)				
Suburban	29,157 (29.4)				
Rural	8,835 (8.9)				
Unknown	3,101 (3.1)				
Income levels, n(%)					
Quintile 1	17,993 (18.2)				
Quintile 2	10,626 (10.7)				
Quintile 3	33,629 (34.0)				
Quintile 4	16,690 (16.9)				
Quintile 5	19,485 (19.7)				
Unknown	645 (0.7)				
Occupation, n(%)					
Dependents of the insured individuals	26,316 (26.6)				
Civil servants, teachers, military personnel and veterans	4,051 (4.1)				
Non-manual workers and professionals	25,388 (25.6)				
Manual workers	35,316 (35.7)				
Other	7,997 (8.1)				
Comorbidity Prevalence, %		**CCI**	**ECI**	**MMI**	**RDCI**
Hypertension	16.79%		V	V	
	13.30%				V
Ulcer or stomach problem	14.19%				V
Other cardiovascular	9.83%				V
Diabetes	7.67%			V	V
Diabetes Uncomplicated (mild to moderate)	6.04%	V	V		
Ulcer disease	4.75%	V			
Coronary heart disease	4.48%			V	
Lung disease	4.22%				V
Chronic Pulmonary Disease	3.69%		V		
Liver Disease	3.57%		V		
Peptic Ulcer Disease excluding bleeding	3.54%		V		
Chronic pulmonary disease	3.15%	V			
Cerebrovascular disease	2.74%	V			
Diabetes with chronic complications	2.58%	V	V		
Chronic obstructive pulmonary disease	2.24%			V	
Cancer	2.18%			V	V
Any tumor	2.05%	V			
Solid Tumor without Metastasis	1.99%		V		
Depression	1.90%			V	
	1.78%				V
Stroke	1.80%				V
	1.03%			V	
Cardiac Arrhythmia	1.76%		V		
Asthma	1.63%			V	
Congestive heart failure	1.29%	V			
Congestive Heart Failure	1.22%		V		
Viral hepatitis	1.14%			V	
Valvular Disease	0.87%		V		

Mild liver disease	0.82%	V			
Other Neurological Disorders	0.8%		V		
Renal Failure	0.78%		V		
Renal disease	0.75%	V			
Chronic Kidney Disease	0.69%			V	
Psychoses	0.65%		V		
Dementia	0.55%	V			
Fracture spine, hip, or leg	0.54%				V
Peripheral vascular disease	0.52%	V			
	0.50%		V		
Hypothyroidism	0.51%		V		
Myocardial infarction	0.49%				V
Fluid and Electrolyte Disorders	0.46%		V		
Deficiency Anemia	0.40%		V		
Paralysis	0.33%		V		
Hemiplegia	0.31%	V			
Metastatic solid tumor	0.30%	V			
Metastatic Cancer	0.30%		V		
Myocardial infarct	0.23%	V			
Alcohol Abuse	0.18%		V		
Blood Loss Anemia	0.12%		V		
Moderate or severe liver disease	0.12%	V			
Weight Loss	0.10%		V		
Obesity	0.10%		V	V	
Coagulopathy	0.09%		V		
Pulmonary Circulation Disorders	0.09%		V		
Lymphoma	0.05%		V		
Diverticulitis	0.03%			V	
Drug Abuse	0.02%		V		
Acquired immune deficiency syndrome	0.01%	V	V		

Abbreviations: Charlson Comorbidity Index (CCI), Elixhauser Comorbidity Index (ECI), Multimorbidity Index (MMI), Rheumatic Disease Comorbidity Index (RDCI).* The same comorbidity has a different prevalence rate is due to the ICD code defined by different formulas of index. (Supplementary table 1).

Table S5. One-year and 5-year mortality analyses of the four comorbidity indexes in matched control group.

**Comorbidity indexes**	**Patient number (%)**	**Mortality rate (per 1000 people)**		**Crude HR (95% CI) for death**		**Age- and sex-adjusted HR (95% CI) for death**	
		1-Year	5-Year	1-Year	5-Year	1-Year	5-Year
CCI							
Low score (0-1)	86,680 (87.5)	5.2	28.8	1.0 (Reference)	1.0 (Reference)	1.0 (Reference)	1.0 (Reference)
High score (≥2)	12,388 (12.5)	16.6	165	3.2 (2.7-3.8)	6.1 (5.8-6.5)	1.7 (1.5-2.1)	2.9 (2.8-3.1)
ECI							
Low score (0-3)	79,584 (80.3)	5.7	29.9	1.0 (Reference)	1.0 (Reference)	1.0 (Reference)	1.0 (Reference)
High Score (≥3)	19,484 (19.7)	10.4	110.8	1.8 (1.5-2.1)	3.8 (3.6-4.1)	1.1 (0.9-1.3)	2.1 (2.0-2.2)
MMI							
Low score (0-1)	68,429 (69.1)	6.2	25	1.0 (Reference)	1.0 (Reference)	1.0 (Reference)	1.0 (Reference)
High score (≥1)	30,639 (30.9)	7.6	92.3	1.2 (1.0-1.4)	3.8 (3.6-4.0)	0.6 (0.5-0.7)	1.7 (1.6-1.8)
RDCI							
Low score (0-2)	75,208 (75.9)	5.9	27.5	1.0 (Reference)	1.0 (Reference)	1.0 (Reference)	1.0 (Reference)
High score (≥2)	23,860 (24.1)	8.9	103.7	1.5 (1.3-1.8)	3.9 (3.7-4.1)	0.7 (0.6-0.9)	1.7 (1.6-1.8)

Abbreviations: Charlson Comorbidity Index (CCI), Confidence interval (CI), Elixhauser Comorbidity Index (ECI), Hazard ratio (HR), Multimorbidity Index (MMI), Rheumatic Disease Comorbidity Index (RDCI).

Table S6. The discriminant capacity of the four comorbidity indexes for the 1- and 5-year survival in matched control group.

Models	1-Year mortality		5-Year mortality	
	Harrell's c-statistics	AIC	Harrell's c-statistics	AIC
Base model	0.737	7,385	0.776	31,791
Base model + CCI	0.836	3,829	0.813	27,854
Base model + ECI	0.817	4,015	0.803	28,767
Base model + MMI	0.779	4,102	0.800	28,857
Base model + RDCI	0.791	4,146	0.795	29,200

Abbreviations: Charlson Comorbidity Index (CCI), Elixhauser Comorbidity Index (ECI), Multimorbidity index (MMI), Rheumatic Disease Comorbidity Index (RDCI); The base model included age, sex, income quartile, urbanization, and occupation groups. The Harrell's c-statistics indicates the prediction models, which are as follows: 0.5 (as well as chance), 0.7–0.8 (acceptable), 0.8–0.9 (excellent), and 0.9–1 (outstanding prediction). The AIC statistics was calculated, and a small AIC indicates the better predictive ability of the model.

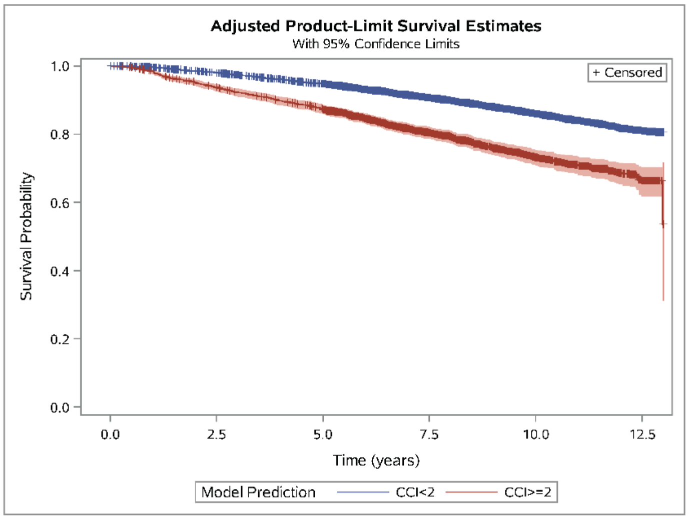

a).

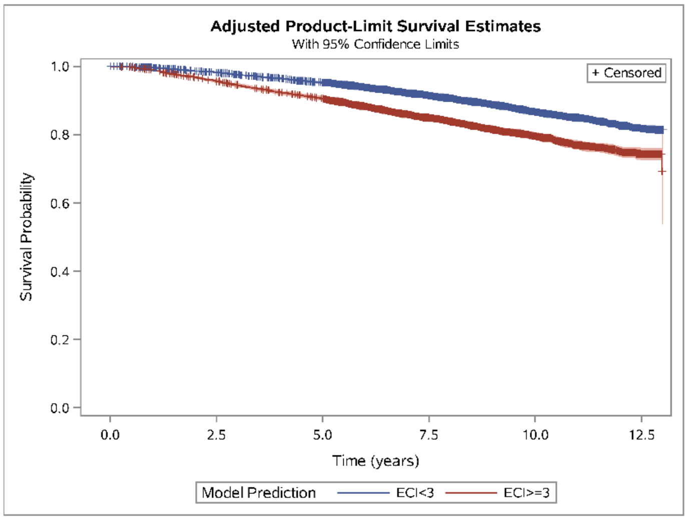

b).

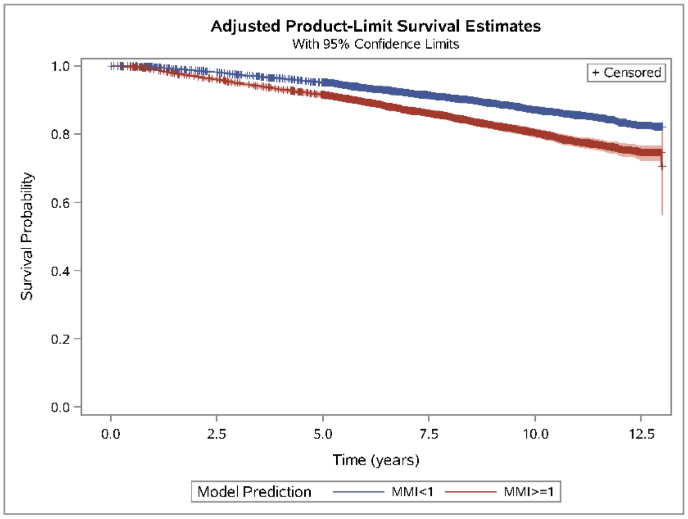

c).

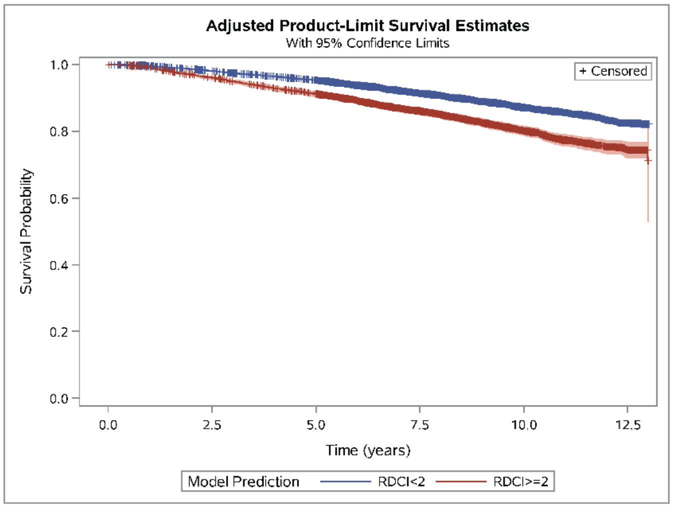

d).

Figure S1. The impact of comorbidity on disease-specific survival showed in weighted Kaplan-Meire curve. (**a**) Charlson Comorbidity Index. (**b**) Elixhauser Comorbidity Index. (**c**) Multimorbidity Index. (**d**) Rheumatic Disease Comorbidity Index. (All log-rank test's p-values <0.001); *The comorbidity indexes are calculated during the diagnostic period (the period of 4 months before and after the initial diagnosis).

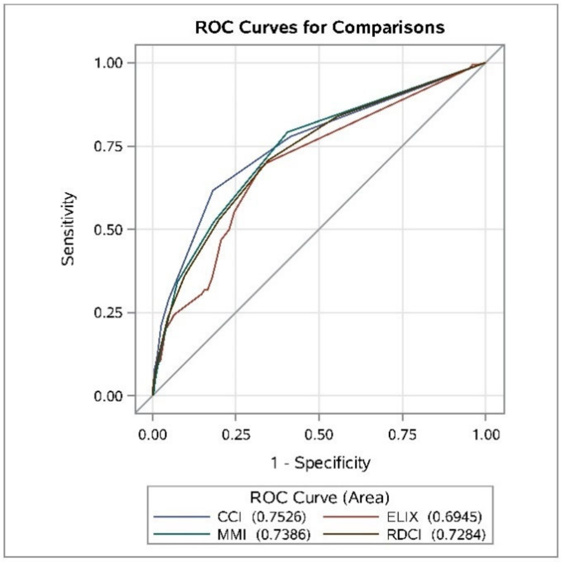

a).

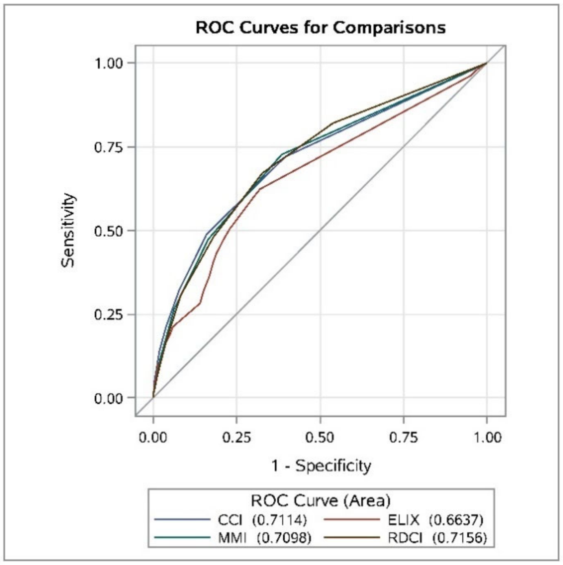

b).

Table S2. Receiver Operating Characteristic (ROC) Curve for Prediction of (**a**) 1-year Mortality and (**b**) 5-year Mortality by Four Comorbidity Indexes.

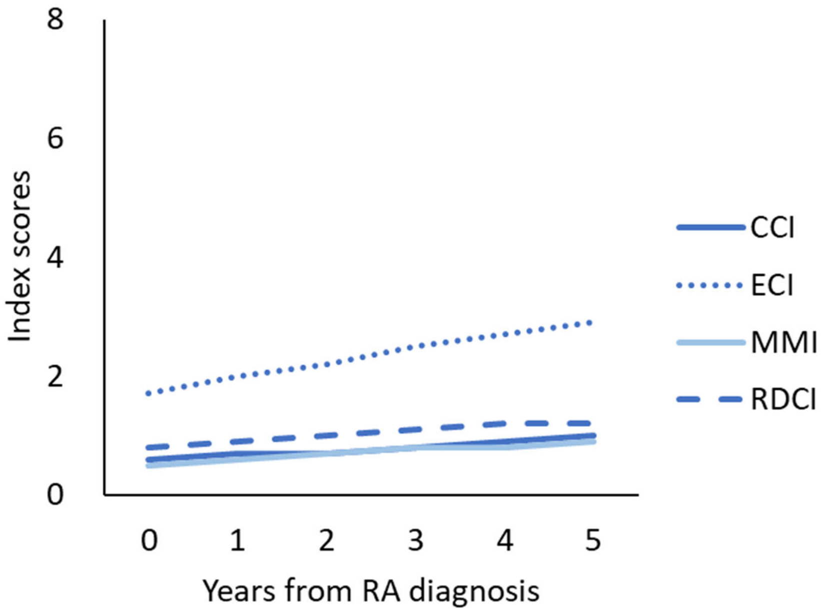

Figure S3. The mean scores of the comorbidity indexes according to the index year in matched control group.

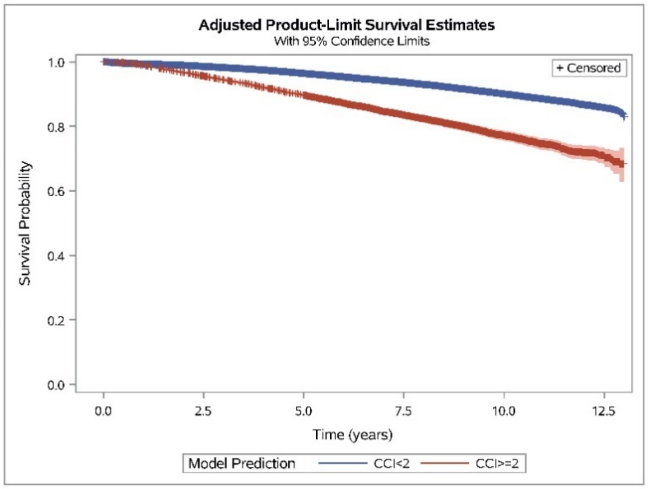

a).

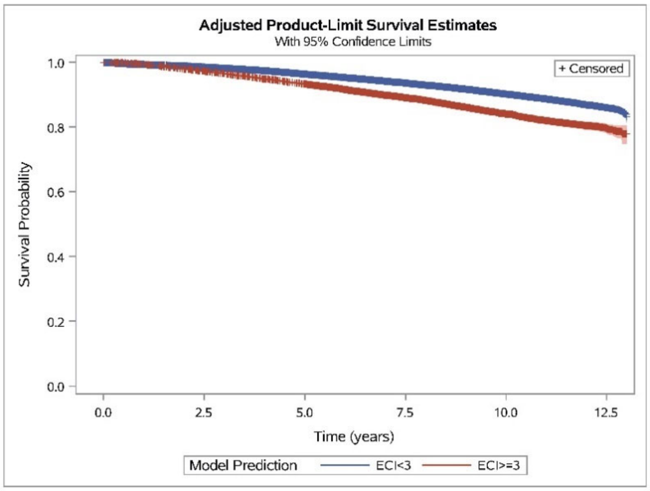

b).

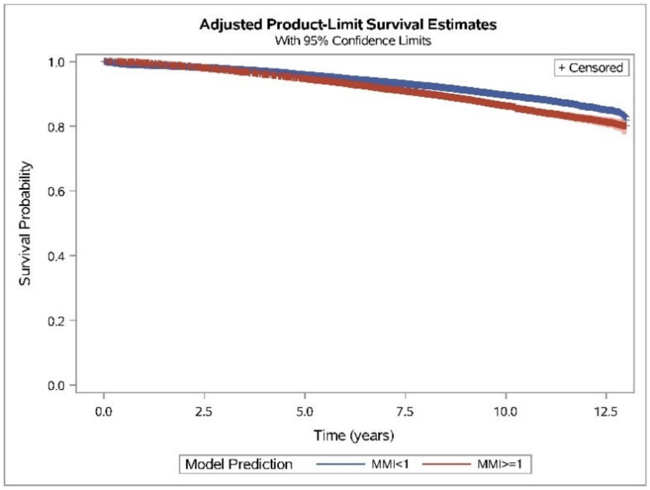

c).

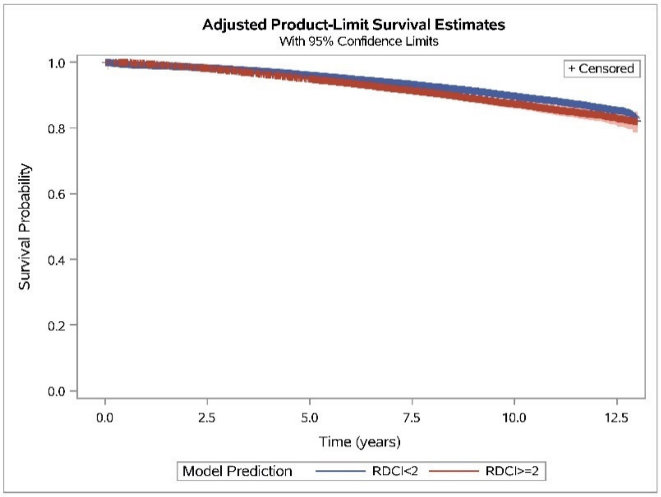

d).

Figure S4. The impact of comorbidity on disease-specific survival in matched control group showed in weighted Kaplan-Meire curve. (**a**) Charlson Comorbidity Index. (**b**) Elixhauser Comorbidity Index. (**c**) Multimorbidity Index. (**d**) Rheumatic Disease Comorbidity Index. (All log-rank test's p-values <0.001).

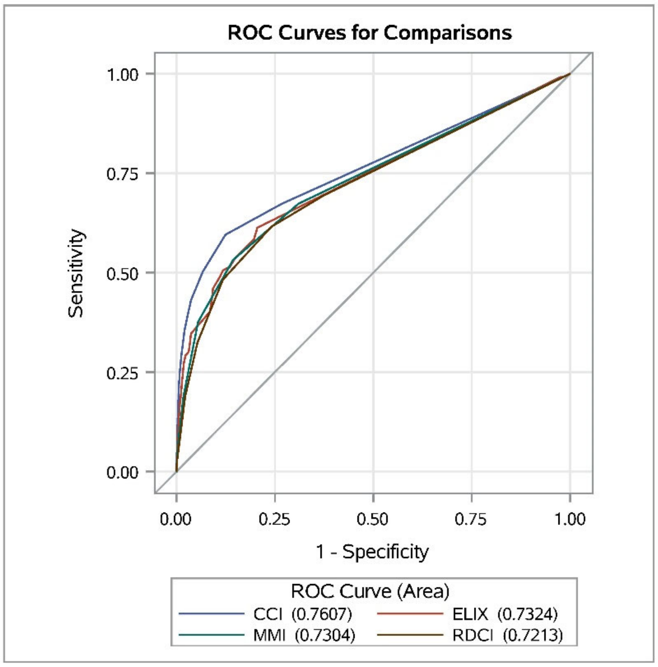

a).

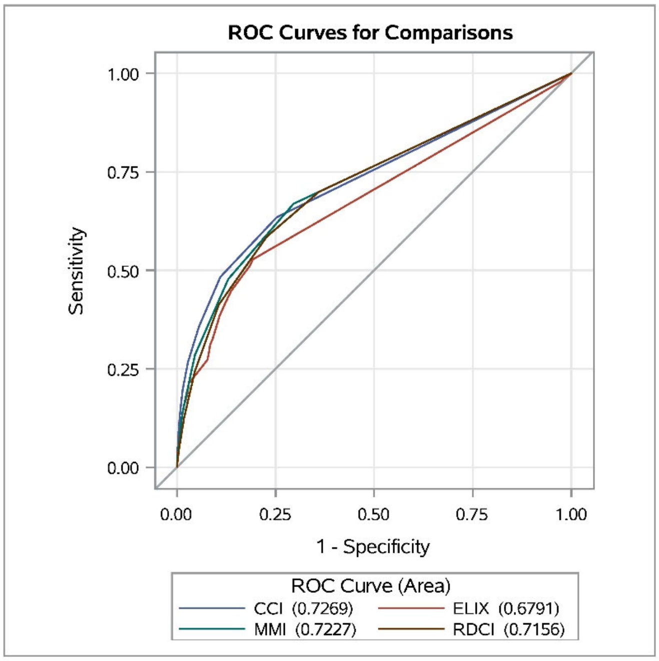

b).

Figure S5. Receiver Operating Characteristic (ROC) Curve for Prediction of (**a**) 1-year mortality and (**b**) 5-year mortality by four comorbidity indexes in matched control group.
